# A systematic review of the efficacy, effectiveness and cost-effectiveness of workplace-based interventions for the prevention and treatment of problematic substance use

**DOI:** 10.3389/fpubh.2022.1051119

**Published:** 2022-11-07

**Authors:** Ashleigh K. Morse, Mina Askovic, Jayden Sercombe, Kate Dean, Alana Fisher, Christina Marel, Mary-Lou Chatterton, Frances Kay-Lambkin, Emma Barrett, Matthew Sunderland, Logan Harvey, Natalie Peach, Maree Teesson, Katherine L. Mills

**Affiliations:** ^1^The Matilda Centre for Research in Mental Health and Substance Use, University of Sydney, Sydney, NSW, Australia; ^2^eCentre Clinic, School of Psychological Sciences, Macquarie University, Sydney, NSW, Australia; ^3^Deakin Health Economics, Deakin University, Geelong, VIC, Australia; ^4^Priority Research Centre for Brain and Mental Health, University of Newcastle, Newcastle, NSW, Australia

**Keywords:** substance use, alcohol use, drug use, workplace, systematic review

## Abstract

Employee alcohol and other drug use can negatively impact the workplace, resulting in absenteeism, reduced productivity, high turnover, and worksite safety issues. As the workplace can influence employee substance use through environmental and cultural factors, it also presents a key opportunity to deliver interventions, particularly to employees who may not otherwise seek help. This is a systematic review of workplace-based interventions for the prevention and treatment of problematic substance use. Five databases were searched for efficacy, effectiveness and/or cost-effectiveness studies and reviews published since 2010 that measured use of psychoactive substances (i.e., alcohol, cannabis, hallucinogens, inhalants, opioids, sedatives, hypnotics, anxiolytics, and stimulants) as a primary or secondary outcome, in employees aged over 18. Thirty-nine articles were identified, 28 describing primary research and 11 reviews, most of which focused solely on alcohol use. Heterogeneity between studies with respect to intervention and evaluation design limited the degree to which findings could be synthesized, however, there is some promising evidence for workplace-based universal health promotion interventions, targeted brief interventions, and universal substance use screening. The few studies that examined implementation in the workplace revealed specific barriers including lack of engagement with e-health interventions, heavy use and reluctance to seek help amongst male employees, and confidentiality concerns. Tailoring interventions to each workplace, and ease of implementation and employee engagement emerged as facilitators. Further high-quality research is needed to examine the effectiveness of workplace substance use testing, Employee Assistance Programs, and strategies targeting the use of substances other than alcohol in the workplace.

**Systematic review registration:**

https://www.crd.york.ac.uk/prospero/display_record.php?RecordID=227598, PROSPERO [CRD42021227598].

## Introduction

Most people who use alcohol and other drugs, both legal and illegal, are employed (1) and 60% of people with substance use disorders (SUDs) have been found to be currently employed (2). Although the relationship is complex, employee substance use has long been associated with a range of negative work-related outcomes including absenteeism (3, 4), loss of productivity (5), high turnover (6), and workplace accidents (7). The nature of a person's work and their workplace may also impact on substance use through factors such as job-related stressors, availability of substances in the workplace environment, and workplace substance use norms (3, 8–11). Of particular concern to employers, workplace stressors may specifically increase substance use that occurs before, during and after work (12).

Irrespective of whether substance use is or is not directly associated with employees' work, the workplace setting offers key opportunities for prevention, early intervention and treatment. In particular, workplace-based initiatives may facilitate early identification of those at-risk of problematic substance use, as well as facilitate access to appropriate supports, thereby reducing the likelihood of adverse personal and occupational outcomes (13). However, the workplace can also be a complex intervention setting due to the influence of both workplace (e.g., workplace culture) and workforce (e.g., age and sex) characteristics on substance use (14–17) and intervention implementation (18).

Recognizing the potential of the workplace as setting for substance use interventions, a growing number of workplace-based initiatives and interventions have been developed and evaluated. Although a relatively large number of studies have been conducted, there is considerable heterogeneity with regard to the samples and interventions investigated, the methods used, and quality of this research undertaken, making it difficult for organizations and practitioners to interpret the findings. Although several reviews have been undertaken they have been limited in scope, focusing on specific population groups (19, 20), intervention modalities (21), study designs (22), particular drug classes (23); and/or are somewhat dated. A rigorous comprehensive review and synthesis of the contemporary literature regarding the efficacy, effectiveness and cost-effectiveness of workplace-based interventions for problematic substance use, including an examination of factors influencing their impact, and barriers and facilitators to implementation, is necessary to help guide organizational decision making and inform future research priorities. As such, this review aims to examine:

i. The efficacy, effectiveness and cost-effectiveness of workplace-based interventions for problematic substance use;
ii. Workforce characteristics that influence the impact of interventions on substance use outcomes; and
iii. Barriers and facilitators to implementing interventions for different drug classes in the workplace.

## Methods

The protocol for this systematic review was prospectively registered with the International Prospective Register of Systematic Reviews (PROSPERO; registration number: CRD42021227598).

### Search strategy

For full details of the search strategy, see Supplementary materials. Five electronic databases of published literature were searched: PsycINFO, MEDLINE, Embase, Cochrane Library and Scopus. A combination of free-text keywords and Medical Subject Headings (MeSH) were adapted to the conventions of each database. Search terms related to three main topics:

1. Substance use (e.g., “substance use,” “alcohol use”);
2. Intervention setting (e.g., “workplace,” “workforce”); and
3. Study design (e.g., “randomized controlled trial,” “cost-effectiveness”)

Following full-text screening, the reference lists of included articles (snowballing) and those articles citing them (reverse snowballing) were searched manually to identify additional eligible articles.

### Inclusion and exclusion criteria

Inclusion criteria were as follows: (i) published in 2010 or later; (ii) written in English; (iii) conducted with human participants; (iv) participants were aged 18 or over; (v) measured the efficacy, effectiveness and/or cost-effectiveness of an intervention; (vi) reported individual-level outcomes relating to the use of alcohol, cannabis, hallucinogens, inhalants, opioids, sedatives, hypnotics, anxiolytics, or stimulants (irrespective of whether the substance was prescribed or non-prescribed); (vii) participants were currently employed and were not volunteers; and (viii) intervention was provided by or sourced through participants' employer (i.e., workplace-based). Articles were excluded if they utilized cross-sectional, cohort or case study designs or did not describe the results of a study (e.g., protocol papers, commentaries, conference abstracts, editorials).

### Data collection

Screening and data extraction were conducted in Covidence, a web-based software platform developed by Veritas Health Innovation, Australia. Following the removal of duplicates, three reviewers independently screened (title and abstract) and conducted full-text review of each article (two reviewers per article; AM, JS, MA); conflicts were resolved via consensus. Two reviewers extracted data from each article, and conflicts were resolved by the third reviewer. One author (MLC) extracted economic evaluation data from relevant articles. Extracted data included study (e.g., setting, target population), participant (e.g., age, sex), and intervention (e.g., offered to all employees or targeted, focused on substance use or broader health and well-being) characteristics, as well as outcomes measured.

### Evidence synthesis and quality assessment

A narrative synthesis approach was used to address the research questions. A modified version of the Downs and Black quality assessment (24) was used to assess the quality of primary studies. Two investigators independently rated each study, and conflicts were resolved by the third reviewer (AM, JS, MA). Quality ratings were as follows: poor (scored ≤14), fair (15–18, 25), good (19, 26–30) and excellent (20–22).

The quality of systematic reviews was assessed using the US National Institutes of Health (NIH) Quality Assessment Tool for Systematic Reviews and Meta-Analyses (31), comprising seven items, with an additional item for reviews that also included a meta-analysis. Higher scores indicated higher quality.

Economic evaluation quality was assessed using the Drummond Checklist (32) comprised of 10 criteria involving 33 sub-questions answered by ‘yes' (scored 1), ‘no' (scored 0) and ‘can't tell' (scored 0.5). Studies that scored at least 9 were considered ‘Good' quality, between 6 and 8 were ‘Fair' quality, and 5 or below were ‘Poor' quality.

## Results

For full details of the characteristics and findings of each primary research study, see Table 1; for reviews, see Table 2.

**Table 1:** Characteristics of primary studies evaluating the effectiveness workplace-based interventions for the prevention and treatment of problematic substance use.

		**Participant characteristics**					
**Study and study design**	**Country and industry**	**Sample (*n*)**	**Study population**	**Intervention and control description**	**Substance use outcomes evaluated**	**Analysis and findings**	**Quality assessment**
Addley et al. (33) RCT	UK Public service	Baseline: 180 12-month follow-up: 132 (73.3%)	White-collar, predominantly full-time employees	Participants were randomly assigned to: Comprehensive group: Multi-component health promotion intervention, including a health risk assessment (screening and normative feedback) and a half-day health and wellbeing session covering alcohol use, smoking, fitness and stress. Participants also completed 3 modules (2 hours each) over 12 months and had access to online resources Limited group: Health risk assessment (screening and normative feedback) only Control group: No treatment. Screening only	Alcohol consumption (units/week)	Between-group analysis showed that there were no significant differences in degree of change from baseline and follow-up between the three groups in alcohol consumption (baseline values were included as a covariate) There were no statistically significant changes in alcohol consumption from baseline to follow-up within any of the three groups	Fair
Joseph et al. (34) Single arm pre-post-test	India Health	Baseline: 39 4-month follow-up: 31 (79.5%)	Hospital manual laborers (blue-collar) that screened positive for moderate-high risk drinking	Workers received screening and an immediate brief intervention (10-20 mins) including normative feedback and advice, followed by a second brief intervention 4 months later	ASSIST score Alcohol consumption	ASSIST scores significantly decreased from baseline (*M* = 26.55) to 4-month follow-up (*M* = 20.06, t = 6.430, p = 0.001). Compared to baseline, significantly fewer participants had high-risk ASSIST scores at follow-up (χ2 = 9.95, df = 1, p = 0.001; 13 high risk participants moved down into the moderate risk category). Additional analyses pertaining to change in relation to individual items of the ASSIST were reported in the study manuscript At 4-month follow-up, there was a significant difference in participant alcohol consumption compared to baseline (χ2 = 17.7, *p* = 0.001) At baseline, 17 participants were daily alcohol users, which decreased to three participants in this category at follow-up	Poor
Brendryen et al. (35) RCT	Norway Multiple industries: health, public service and consulting	Baseline: 85 2-month follow-up: 56 (65.9%) 6-month follow-up: 63 (74.1%)	Hospital workers, public administration, teachers, social workers, and consultants/IT workers that screened positive for risky drinking. Mainly white-collar	Workers that screened positive for risky drinking received personalized feedback and then randomly assigned to: Intensive treatment group: Multi-component alcohol harm minimization intervention (62 online sessions lasting 3-10 mins). Sessions were conducted online in an interactive format and focused on goal setting and tracking alcohol consumption, relapse prevention, emotion regulation, and alcohol education. Brief intervention (control) group: Alcohol information e-booklet	Alcohol consumption (units/week)	Compared to baseline, participants randomized to receive intensive treatment consumed significantly fewer drinks per week at 2-month follow-up *M* = 17.0 v *M* = 13.7; t (42) = 2.29, p =.03 and at 6-month follow-up (*M* = 17.0 v *M =* 13.4; t (42) = 3.42, *p* < 0.001) Compared to baseline, participants randomized to receive the brief (control) intervention consumed significantly fewer drinks at 6 months (*M* = 17.3 v *M =* 14.6; *t* (41) = 3.84, *p* < 0.001). Drinks per week at 2 months (*M* = 16.1) did not significantly differ from baseline in the control group. Intention-to-treat analyses did not find significant between group differences at each time point. Regression analyses of complete cases in each group only, controlling for baseline scores showed a significantly significant difference between groups, with those in the intervention group drinking less than those in the control group at 2 months (B = 5.68, 95% CI [0.48–10.87], *p* = 0.03)	Good
Broome et al. (36) RCT (cluster randomized)	US Hospitality	Baseline: 235 6-month follow-up: 190 (80.9%) 12-month follow-up: 147 (62.6%)	Young restaurant workers in a restaurant chain	Restaurant sites were cluster randomized to: Treatment group: Three educational alcohol harm minimization sessions adapted from the 'Team Awareness' training program (2 hours each session) Control group: No treatment. Screening only	Proportion reporting any heavy drinking (consumed ≥5 drinks in a session on ≥1 day of the past 30 days) Proportion reporting recurring heavy drinking (heavy drinking on ≥5 days of the past 30 days)	The treatment group did not differ from the control group in changes in the proportion reporting any heavy drinking from baseline (60 vs. 60%) to the average of 6-month and 12-month follow-ups, or from 6-month (64 vs. 72%) to 12-month (47 vs. 64%) follow-up The treatment group had significantly larger reductions in the proportion reporting recurring heavy drinking than the control group when comparing baseline (35 vs. 26%) to the average of 6- and 12-month follow-ups (B = −0.690, z = −1.97, *p* = .049). The adjusted odds of recurring heavy drinking in the treatment group during the follow-up period were approximately half what they had been before training (OR = 0.49). The treatment group did not differ from the control group in changes in recurring heavy drinking from 6-month (34 vs. 43%) to 12-month (20 vs. 38%) follow-up	Poor
Burgess et al. (37) Quasi-experimental pre-post-test	Russia Manufacturing	Baseline: 66 (treatment) 337 (control) 90-day follow-up (treatment): 66 (100%) 120-day follow-up (control): 338 (100.3%)	Industrial workers	Workers screening positive for drinking problems at a company with an EAP (treatment group) were compared with the general population of workers those from a company with no EAP (control group): Treatment group: Employee Assistance Program (the exact treatment services received differed for each individual and was not well-described) Control group: No treatment. Screening only	AUDIT scores	AUDIT scores in the treatment group reduced from baseline to follow-up (M = 13.79 v M = 3.76). AUDIT scores in the control group remained low and stable over time from baseline to follow-up (M = 3.6 v M = 3.8) The reductions observed in the treatment group were significantly greater than the control group (F = 145.630 (1, 803), *p* < 0.000).	Poor
Burnhams et al. (38) RCT (cluster randomized)	South Africa Public Service	Baseline: 325 3-month follow-up: 189 (58.2%)	Employees of two public service divisions: one responds to emergency situations and the other upholds law and order	Employees were cluster randomized to: Treatment group: Educational alcohol harm minimization intervention adapted from the 'Team Awareness' training program (8-h session) Control group: Short educational intervention (1-hour wellness talk)	Binge drinking (days in the past 30 days participant consumed ≥5 drinks in one sitting) Problematic drinking (CAGE)	There was a significant group by time interaction for binge drinking, F (1.117) = 25.16, *p* < 0.0001. Participants in the treatment group reduced past 30-day binge drinking from 2.1 days to 1.4 days at follow-up. Participants in the control group increased binge drinking days from 1.6 days at baseline to 2.1 days at follow-up There were no significant group by time interaction or main effects for group or time for CAGE scores	Good
Goetzel et al. (39) Prospective pre-post-test	US Multiple industries: including education, trade, social services, public administration, healthcare	Baseline: 5,362 1-year follow-up: 2,458 (45.8%)	Employees of small employers in a range of industries	Workers were assigned to a multi-component health promotion intervention (included a health risk appraisal, online resources, and phone-based health coaching)	Prevalence of high-risk alcohol consumption ROI analysis Cost categories: - Program costs, medical treatment costs, wage Outcomes: - Medical and productivity savings	There was a statistically significant reduction in the prevalence of high-risk alcohol consumption from baseline compared to 1-year follow-up (8.34 vs. 6.59%; *p* ≤ 0.001). Projected medical savings were US$124,867 and productivity savings of US$310,040 from reduction in 10 health risk behaviors. Cost of the program was $214,347. Total ROI is $2.03 for every dollar invested	Poor
Gómez-Recasens et al. (40) Single arm pre-post-test	Spain Construction	Baseline: 1103 Year 1 follow-up: 990 (89.8%) Year 2 follow-up 700 (63.5%) Year 3 follow-up: 625 (56.7%)	Industrial workers in Spain (mainly blue-collar)	Workers were assigned to receive Step (1): Company-wide substance use education (e.g., brochures, training). In addition, medical examination interviews and drug tests were conducted to monitor employee substance use. Participants who were consuming risky amounts of alcohol, or consuming drugs were assigned to additionally receive Step (2): secondary prevention including brief intervention (10-15 mins), personalized advice or referral to specialist services	Risky alcohol use (as assessed by the semi-structured interview) Risky drug use (positive drug test) Total risky use (combination of risky drug and alcohol use)	A significant decrease in the prevalence of risky alcohol use was observed from baseline to year 1 (14.6 vs. 10.6%; p < 0.001). This initial decrease was maintained at year 2 (9.3% vs baseline, *p* < 0.001 and year 3 (10.7% vs. baseline, *p* < 0.001). The prevalence of risky drug use increased significantly from year 1 to year 2 (6.7 vs 6.9%; p =.039), but not at any other points The prevalence of total risky use significantly reduced from baseline to year 1 (19 vs. 15.3%; *p* < 0.001). This initial decrease was maintained at year 2 (year 2: 14.4% vs baseline, *p* < 0.001) and year 3 (14.9% vs. baseline, *p* < 0.001)	Poor
Harada et al. (41) RCT	Japan Not specified	Baseline: 86 1-month follow-up: 83 (96.5%) 3-month follow-up: 83 (96.5%)	Workers (occupations not specified) that screened positive as heavy drinkers but were not alcohol dependent	Workers were randomized to: Treatment group: Multi-component alcohol harm minimization intervention (same as the comparison group), with the addition of stress management education, calendar-based self-monitoring, interactive follow-up via email and phone and group discussions Comparison group: Multi-component alcohol harm minimization intervention (assessment, normative feedback, three workbook-based educational sessions with a nurse, and blood tests)	Mean daily alcohol consumption AUDIT scores	Mean daily alcohol consumption decreased significantly over time in the treatment group (baseline: 5.27, 1 month: 4.25, 3 months: 3.82; *p* < 0.001) and the comparison group (baseline: 5.22, 1 month: 3.96, 3 months: 3.70; *p* < 0.001). There were no significant between-group differences in this change. AUDIT scores decreased significantly over time in the comparison group (baseline: 14.7, 1 month: 13.4, 3 months: 12.7; *p* =.022), but not in the treatment group (baseline: 13.3, 1 month: 12.4, 3 months: 12.2; *p* =.136). There were no significant differences in the change in AUDIT scores between groups	Good
Hermansson et al. (42) RCT	Sweden Transport	Baseline: 194 12-month follow-up: 158 (81.4%)	Transport workers (~50% were manual laborers) that screened positive as harmful drinkers	Workers were randomly allocated to: Comprehensive group: A choice of up to 3 different sessions. The first session was the same brief intervention delivered to the limited group. The second session asked participants to systematically recall their alcohol consumption over the past 14 days in a TLFB interview. The third session asked participants to keep a daily self-monitoring drinking diary over 4 weeks Limited group: Brief intervention (15 mins) consisting of assessment, normative feedback and written advice Control group: No treatment. Screening only	AUDIT scores CDT levels (alcohol biomarker) Proportion screening positive on the AUDIT for harmful use (scores ≥8) Proportion screening positive on the CDT test	The three groups did not differ significantly in changes from baseline to 12 months in AUDIT score or in CDT levels When all three study groups were combined, the overall proportion of participants screening positive for harmful alcohol use significantly decreased from 51.3% at baseline to 22.8% at follow-up (*p* < 0.0001). For biomarker results, 57.6% tested positive at screening compared with 34.2% at follow-up (*p* < 0.0001)	Good
Ito et al. (43) RCT	Japan Not specified	Baseline: 296 3-month follow-up: 282 (95.3%) 12-month follow-up: 277 (93.6%)	Employees (mostly blue-collar) of large companies that screened positive as heavy drinkers but were not alcohol dependent	Workers were randomized to: Comprehensive group: 2 brief intervention sessions (15 mins each) and completion of a drinking diary Limited group: 2 brief intervention sessions (15 mins each) Control group: Alcohol information booklet	Total drinks (past 7 days) Number of binge drinking episodes (past 28 days; binge drinking defined as drinking >60 g of alcohol/day) Number of alcohol-free days (past 28 days)	Significant reductions in mean total drinks were observed between baseline and 3-month and baseline and 12-months for all three groups (*p*'s < 0.05). The change over time in total drinks did not significantly differ between groups. Comprehensive group: baseline: *M* = 35; 3 months: *M* = 28.8; 12 months: *M* = 27.5 Limited group: baseline: *M* = 35.2; 3 months: *M* = 29; 12 months: *M* = 24.1 Control group: baseline: *M* = 32.5; 3 months: *M* = 29.1; 12 months: *M* = 25.5 The comprehensive and limited groups demonstrated significant reductions in mean binge drinking episodes from baseline to 3 months (*p*'s < 0.01) and baseline to 12 months (*p*'s < 0.005). The control group did not demonstrate significant change in binge drinking episodes over time. Change in binge drinking episodes did not significantly differ between the groups Comprehensive group: baseline: *M* = 8.33; 3 months: *M* = 5.69; 12 months: *M* = 4.68 Limited group: baseline: *M* = 7.55; 3 months: *M* = 5.15; 12 months: *M* = 4.44 All three groups demonstrated significant increases in mean alcohol-free days from baseline to 3 months (*p*'s < 0.01) and baseline to 12 months (*p* < 0.05). Change in alcohol free days from baseline was significantly different between groups at 3 months (*p* =.008) and at 12 months (*p* = 0.021). Improvement in alcohol-free days was significantly greater in the limited group (but not the comprehensive group) compared to the control group at 3 months and 12 months (ps < 0.05). Comprehensive group: baseline: *M* = 4.11; 3 months: *M* = 5.78; 12 months: *M* = 7.46 Limited group: baseline: *M* = 4.63; 3 months: *M* = 7.97; 12 months: *M* = 8.95 Control group: baseline: *M* = 5.00; 3 months: *M* = 6.25; 12 months: *M* = 6.58	Fair
Jones et al. (44) Quasi-experimental pre-post-test	UK Military	Baseline: 103 Follow-up (time varied widely, up to 36 months): 83 (80.6%)	Reserve forces military personnel being treated for mental health problems attributable to operational deployment	Eligible workers were categorized into two groups based on treatment history: Treatment group: Reserve military personnel who accessed the Reserves Mental Health Programme (RMHP), a program available to demobilized personnel with concerns about their mental health. Based on results of a formal assessment, treatment is offered by defense mental health service personnel Control group: Military personnel who did not receive treatment	Proportion of participants qualifying for AUDIT caseness (scores ≥8)	Controlling for baseline AUDIT scores, those in the treatment group did not significantly differ in the change over time in AUDIT caseness compared to the control group. No within group analyses were reported Treatment group: baseline: 70.0%; follow-up: 45.7% Control group: baseline: 30.8; 28.6%	Poor
Khadjesari et al. (45) RCT	UK Not specified	Baseline: 1,330 3-month follow-up: 1,066 (80.2%)	Employees (scoring ≥5 on the AUDIT) of a large company. Most workers were in management	Workers screening positive as harmful drinkers were randomized to: Treatment group: Received feedback on all health behaviors assessed in a health risk appraisal (including alcohol) and referral to an online resource, Down Your Drink (DYD), a multi-component alcohol harm minimisation intervention Control group: Received feedback on all health behaviors except alcohol consumption	Alcohol outcomes Self-reported past week alcohol use (TOT-AL; UK alcohol units per week) AUDIT score	There were no significant differences between the control and treatment group in past week alcohol consumption (*M* = 19.06 v *M* = 20.25) or AUDIT scores (*M* = 6.26 v *M* = 6.26) at 3-month follow-up. No within group analyses were reported	Good
Kouwenhoven-Pasmooij et al. (46) RCT (cluster randomized)	Netherlands Military, law enforcement and health	Baseline: 491 6-month follow-up: 354 (72%) 12-month follow-up: 324 (66%)	Military, police and hospital workers that screened positive for risk of developing cardiovascular disease	Workers were cluster randomized to: Treatment group: Web-based health risk assessment with advice, as well as 7 individual coaching sessions with an occupational health physician Control (limited) group: Web-based health risk assessment with advice	Proportion of participants with excessive alcohol use (>7 [women] or >14 [men] glasses of alcohol consumed per week)	Proportion of the treatment group using alcohol excessively significantly decreased from baseline (14%) to 6-months (8.9%, *p* < 0.05) and baseline to 12 months (4.6%, *p* < 0.05). Proportion of the control (limited) intervention group using alcohol excessively significantly reduced from baseline (15.9%) to 12-months 4.8%, p < .05, but not to 6 months. The proportion of participants with excessive alcohol use at 6-month or 12-month follow-ups did not differ between groups	Good
Kuehl et al. (47) RCT (cluster randomized)	US Law enforcement	Baseline: 408 6-month follow-up: 352 (86.3%) 12-month follow-up: 311 (76.2%) 24-month follow-up: 313 (76.7%)	Law enforcement personnel from police and sheriff departments	Workers were cluster randomized to: Treatment group: Educational health promotion intervention (SHIELD program), receiving 12 group sessions (30 mins each) over 6 months Control group: Assessment and normative feedback	Alcohol consumption (Health Maintenance Consortium)	In alcohol consumption, the treatment group significantly differed from the control group in change from baseline to 12 months (effect size = 0.16, *p* < 0.05). The treatment group did not significantly differ from the control group in changes from baseline to 6 months, 24 months, or to the average of all three follow-up points. No within-group analyses were reported	Poor
LeCheminant et al. (48) Single arm pre-post-test	US Education	Baseline: 2,398 Analyzed (those who reported drinking alcohol at baseline with complete data at 1- and 2-year follow-ups): 691 (28.8%)	Public school workers (mostly abstinent or low-risk drinkers)	Workers received a multi-component health promotion intervention (including assessment, education and exercises around diet and exercise)	Alcohol consumption (drinks per day)	Mean drinks per day significantly decreased from baseline (1.31) to the 1-year (1.16) and 2-year (1.10) follow-up period (*F* = 30.0, *p* < 0.0001). Note: reported statistics include only those who reported drinking alcohol (*n* = 691)	Poor
Michaud et al. (49) RCT	France Not specified	Baseline: 787 12-month follow-up: 435 (55.3%)	Office workers that screened positive for hazardous drinking but were not alcohol dependent	Workers were randomly allocated to: Treatment group: Brief intervention including normative feedback and advice Control group: Alcohol information and self-help booklet	Alcohol consumption (grams per week) AUDIT score Proportion of participants reducing AUDIT scores below at-risk threshold (7 for men and 6 for women) Alcohol biomarkers Mean corpuscular volume (MCV) Gamma-glutamyl-transferase (GGT) Covariates Age Gender	Reductions in alcohol consumption from baseline to 12-months were significantly greater in the treatment group (−60 grams/week) compared to the control group (−44 grams/week; p =.038). Reductions in mean AUDIT scores from baseline to 12-months were significantly greater in the treatment group than the control group (−1.51 vs. −0.71; *p* = 0.009). In addition, mean AUDIT scores at 12-months were significantly lower in the treatment group compared to the control group (6.59 vs. 7.55; *p* = 0.01). The same pattern of results were found in analyses by sex, but the treatment group effect was only significant in males There were no significant differences between groups in the proportion of participants reducing AUDIT scores below at-risk threshold or in alcohol biomarkers Younger ages were highly associated with greater reductions in AUDIT scores (*p* < 0.0001) and alcohol consumption (*p* < 0.0001)	Fair
Pidd et al. (50) Non-randomized study	Australia Manufacturing	Baseline: 450 12-month follow-up: 264 (58.7%) 24-month follow-up: 275 (61.1%)	More than 80% of participants were blue collar employees, the remaining were white-collar	Two worksites were allocated to the treatment group and two were allocated to the control group (non-random assignment): Treatment group: Multi-component alcohol harm minimization intervention (including co-designed workplace alcohol policy, education, exercises, and referral program) Control group: No treatment. Screening only	AUDIT-C score	AUDIT-C scores did not significantly change over time from baseline to 12 months, or from baseline to 24 months in either group At 12 months, the mean AUDIT-C score in the treatment group, 4.1 was significantly higher than the mean in the control group, 2.6 (*p* = 0.002). At 24 months, AUDIT-C scores were not significantly different between the groups. Note: additional statistics for comparisons regarding specific AUDIT-C items were reported in the study manuscript	Poor
Reynolds et al. (51) RCT (cluster randomized)	US Not specified	Baseline: 1,382 1-month follow-up: 1046 (75.7%) 6-month follow-up: Final: 870 (63.0%)	Small business employees in industries identified as high risk for alcohol and/or drug abuse in a national survey	Workers were cluster randomized to: Treatment group A: Educational substance use harm minimization intervention (4-hour session; ‘Team Awareness for Small Business'). Designed to decrease substance use by addressing social alcohol use, stress and to enhance help seeking behavior Treatment group B: Educational health promotional intervention (4-hour session, “Choices”) which is tailored for each workplace. Modules could include information regarding tobacco use, prescription drug use, healthy eating, alcohol use Control group: No treatment. Screening only	Alcohol-related outcomes (measured at baseline and 6 months only) Frequency of alcohol use in the past 30 days - continuous coding (0–30) - categorical coding (1–5 days, 6–15 days, 16–21 days, 22–30 days) Occurrence of alcohol-induced workplace incidents: (dichotomous; working under the influence, working with a hangover, missing work because of hangover, or alcohol use affecting work in any way)	Analyses of all participants There was a marginally significant group by time interaction for drinking frequency (continuous), F = 2.95, *p* = 0.05. The mean number of drinks consumed over the past 30 days significantly reduced from baseline to 6-month follow-up for those in treatment group A (3.79 v 2.99; *p* < 0.05), and those in treatment group B (4.56 v 3.87; *p* < 0.05) but did not in the control group (2.72 vs. 2.51). The same pattern of results was evidenced when frequency of alcohol use (categorical coding) was examined (F = 3.22, *p* = 0.04). There were no significant differences in the changes in alcohol-induced workplace incidents from baseline to 6 months between the three groups Alcohol and other drug (AOD) users sub-group analyses Analyses of a subgroup of AOD users only yielded nonsignificant group by time interactions and main effects for alcohol use frequency and for workplace incidents	Poor
Richmond et al. (52) Prospective, quasi-experimental, pre-post-test	US Public service	Baseline: 579 Follow-up (treatment: *M = 3.67* months; control: *M =* 7.90 months): 344 (59.4%)	State government workers that self-selected into EAP services	The treatment group comprised of workers accessing their workplace EAP services were compared with a propensity score matched control group who did not access EAP services: Treatment group: Employee Assistance Program (treatment received was determined by the treating clinician at the program) Control group: No treatment. Screening only	AUDIT score	There were no significant within- or between-group differences in AUDIT scores across the study period Treatment group: baseline: 3.49; follow-up: 3.35 Control group: baseline: 3.54; follow-up: 3.31	Fair
Schouw et al. (53) Single arm pre-post-test	South Africa Energy production	Baseline: 156 2-year follow-up: 137 (87.9%)	Employees of a commercial power plant, (blue-collar and white-collar employees)	Workers received a multi-component health promotion intervention (including assessment, normative feedback, education, food and exercise classes, and leadership modeling positive behaviors)	Proportion of participants engaging in harmful alcohol use (AUDIT score ≥8)	Proportion of participants engaging in harmful alcohol use significantly decreased from baseline to the 2-year follow-up 21 vs 4.8%; *p* = 0.001	Poor
Spicer et al. (54) Prospective controlled pre-post-test	US Railroad	Baseline: 318 Follow-up (treatment: average of 8.7 months; control: average of 10.6 months): 186 (58.5%)	Young railroad workers (18-29 years old)	The treatment group comprised of workers who had attended the PREVENT program, while the control group comprised of workers who has not attended: Treatment group: Educational health promotion intervention (PREVENT) that primarily focused on alcohol and other drug use (2 days) Control group: No treatment. Screening only	Primary outcome: Drinks consumed in the past 30 days (days of alcohol use x drinks per drinking day) Secondary alcohol outcomes (past 30 days): - days of alcohol use - drinks per drinking day - days consuming ≥5 drinks	Controlling for baseline demographic characteristics, smoking and drinking levels the treatment group demonstrated significantly greater reductions in drinks consumed in the past 30 days (-3.1 v 1.2; *RR =* 0.44, 95% CI:0.23, 0.85) and in number of days of alcohol use from baseline to follow-up in the treatment group (−0.6 v 0.4; *RR =* 0.68, 95% CI: 0.50–0.93) Changes in the other alcohol-related outcomes did not significantly differ between groups	Poor
Tinghög (55) Quasi-experimental pre-post-test	Sweden Finance and insurance	Baseline (for alcohol questions): 400 6-month follow-up: 314 (78.5%) 12-month follow-up: 306 (76.5%)	Young finance and insurance salespeople at a company. Mostly white collar	The treatment group comprised of workers at a company that received an education program, while the control group comprised of workers at a company that had not: Treatment group: Educational alcohol harm minimization intervention, consisting of two lectures (45 mins each). Lectures aimed to provide information regarding alcohol risks, change risky alcohol use patterns, and prevent future harmful alcohol use Control group: No treatment. Screening only	AUDIT score Frequency of binge drinking (derived from AUDIT)	The treatment group did not significantly differ from the control group in changes over time in AUDIT scores or binge drinking Within-group analyses were not reported The findings of subgroup analyses comparing outcomes for men versus women, and low consumption (< 6.4 grams of alcohol/day) vs high consumption (≥6.4 grams of alcohol/day) groups were consistent with main findings	Fair
Tinghög and Tinghög (56) Quasi-experimental pre-post-test	Sweden Public service	Baseline: 529 6-month follow-up: 263 (49.7%)	Public sector employees in various areas, including schools, social services, and administration	The treatment group comprised of workers that received an education program, while the control group comprised of workers that received the program after follow-up: Treatment group: Educational alcohol harm minimization intervention, consisting of two half-day sessions Control group: No treatment. Screening only	AUDIT score AUDIT-derived outcomes Frequency of drinking Frequency of binge drinking Typical amount consumed per drinking occasion	The treatment group did not significantly differ from the control group in changes over time in overall AUDIT scores or in the three AUDIT-derived outcomes. Controlling for age and gender did not significantly alter the results Subgroup analyses of low consumption (≤4.14 grams of alcohol/day) versus high consumption groups (≥6.4 grams of alcohol/day) found no significant between-groups difference in change over time in AUDIT scores or AUDIT-derived outcomes for low consumption participants. In high consumption participants, there was a group by time interaction for frequency of drinking (*F* = 6.5, η2 = 0.13, *p* < 0.05), where those in the treatment group demonstrated a decrease in frequency from baseline to follow-up (M = 1.4 to M = 1.0) whereas those in the control group demonstrated an (M = 0.8). There were no significant group by time interactions in high consumption participants for the other three alcohol outcomes	Fair
Watson et al. (57) RCT	UK Public service	Baseline: 55 6-month follow-up: 55 (100%)	Public service council workers who are hazardous drinkers. Even mix of white- and blue-collar	Workers that screened positive for hazardous drinking were randomly allocated to: Treatment group: Brief intervention including normative feedback and advice Control group: No treatment. Screening only	AUDIT score Alcohol consumption (7-day TLFB): - number of days drinking - maximum units of alcohol consumed in one day -total weekly consumption Economic outcomes Economic indicators (service use, employment outcomes, public sector resource and employment costs)	Group by time interactions for all alcohol related outcomes were not significant. Both groups demonstrated a significant reduction in mean AUDIT scores from baseline to 6-month follow-up (*F* = 8.84, *p* = 0.004). Treatment group: baseline: 8.88; follow-up: 7.44 Control group: baseline: 8.76; follow-up: 7.54 There were no significant main effects for any other alcohol consumption outcomes The differences in service costs was calculated at Â£344.5 per person; that is there is a net saving of health and other care costs in the intervention group compared to the control. The QALYs fell in both intervention and control but rather less for the intervention group. The difference is 0.002 (0.010) yields a net advantage of the intervention of 0.008 QALYs	Good
Wierenga et al. (58) Process and effectiveness evaluation	Netherlands University and university hospital	Baseline: 406 Final: 145 (35.7%)	Employees in the Department of Gynecology at a hospital, and the Health Faculty of a university (majority female)	Workers were allocated to: Treatment group: Multi-component health promotion intervention. Focuses of the program included physical activity, smoking, alcohol use, nutrition, and relaxation Control group: No treatment. Screening only	Alcohol consumption (glasses/week)	No significant difference between treatment and control groups, or between baseline and follow-up in either group	Poor

AUDIT, Alcohol Use Disorders Identification Test; CDT, carbohydrate-deficient transferrin in serum; TLFB, Timeline Followback; SHIELD, Safety & Health Improvement, Enhancing Law Enforcement Departments; EAP, Employee Assistance Program; QALY, Quality-adjusted life year; PREVENT, Personal Responsibility and Values: Education and Training; ROI, Return on Investment.

**Table 2:** Characteristics of reviews evaluating the effectiveness workplace-based interventions for the prevention and treatment of problematic substance use.

**References**	**Focus**	**Substance(s)**	**Number of studies**	**Meta-analysis?**	**Main findings**	**Quality assessment**
Akanbi et al. (23)	Employer-led interventions to reduce the adverse effects of drug misuse in the workplace	Drugs (opioid focus)	Twenty seven studies	No	Akanbi et al. (23) reported that most studies reviewed were methodologically weak (rating them all as fair or poor using the ‘Downs & Black criteria') and provided a poor evidence base to examine the efficacies of the interventions. The reviews findings indicate that workplace interventions may be most effective for reducing work-related injuries or accidents (3 of 4 studies using combined interventions reduced workplace injuries or accidents, 5 of 7 studies using drug testing reported it might reduce workplace injuries and one study evaluating the impact of an EAP program led to a decline in workplace injuries). Akanbi et al. (23) suggested workplace injury data may be more reliable due to standard documentation of reporting whilst data on drug use is reliant on self-reports. Overall, this review found mixed results with interventions working in some environments and not others. A meta-analysis was not conducted due to the studies differences in designs, effects measures and outcomes	5/7
Burnhams et al. (25)	Workplace substance abuse prevention programmes that also address substance-related HIV risks in South Africa	Alcohol (majority of included studies)	Fourteen studies	No	A high variability in design, methodology and limited descriptions of methods to ensure intervention integrity was noted. All studies used self-report measures, with seven showing significant reductions in self-reported problem drinking and going to work with a hangover (59), drinking and/or heavy (binge) drinking days in the past month (60–65) because of the intervention (compared to control groups) The review also noted the use of indirect approaches for delivering substance use prevention messages to be less threatening to the corporate sector, such as integrating into employee health and wellness. No notable difference in the effectiveness of direct versus indirect was highlighted by the authors.	6/7
Coenen et al. (22)	Worksite health promotion programs focusing on increasing physical activity, behavior dietary behaviour, reducing alcohol use, and smoking cessation	Alcohol	Fifteen studies (data harmonized)	Yes	Coenen et al. (22) focused on meta-analysis of individual participant data (IPD) from Dutch studies only. Seven studies (*n* = 44,007) reported the effects on alcohol intake, these were included measures in general health and lifestyle interventions. The meta-analysis found health promotion programs in the workplace do not significantly affect alcohol intake (units of alcohol consumed per week, analyzed as z-scores). All alcohol intake studies examined face-to-face interventions, three also included web-based interventions and one included environment interventions. There were no differences found between high and low compliance with the program or socioeconomic position (SEP) on alcohol intake, (compliance was low in the studies on average 51%). In general the meta-analysis review found that workplace health promotion programs were not effective	7/8
Kolar et al. (26)	Two systematic reviews: - review 1 examined alcohol interventions in the workplace - review 2 focused on community sport alcohol interventions	Alcohol	Eighteen studies (workplace review, not including studies in sport review)	No	The four types of interventions identified were brief interventions, web-based interventions, psychosocial interventions and random workplace drug and alcohol testing. Brief interventions were the most frequently implemented and found to provide inconsistent outcomes, yielding non-significant results or only significant for one variable (I.e negative alcohol related consequences, hangover etc.) A limited review of the web-based interventions was provided by Kolar (2015) reporting only that they found significant reductions in alcohol consumption. Psychosocial interventions were favorable but indicated that interventions are more effective if tailored to each workplace. The implementation of random drug and alcohol testing was an effective intervention in decreasing workplace injuries, however, showed no significant reductions in alcohol consumption. This review highlighted the need for robust methodology, longitudinal data from interventions implemented at multiple organizational levels and with sufficient follow-up periods	4/7
Lee et al. (18)	Interventions for risky alcohol consumption among workers within male-dominated industries	Alcohol	Eight studies	No	This review highlights the advantages of implementing intervention and secondary prevention activities for alcohol disorders within the workplace setting. If targeted at risky drinkers, interventions that were low-intensity and use screening had some impact on risky alcohol consumption. Low-intensity multi-modal workplace interventions may be effective in reducing absenteeism in male-dominated workplaces. Lee et al. (18) found that workplace alcohol and drug testing had no clear benefit to reducing harms in the workplace. The review also identified the difficulty in assessing interventions developed for specific workplaces and generalizing to other work force settings	7/7
Mewton et al. (28)	Review of reviews focusing on universal prevention strategies for alcohol and illicit drugs, including family, school, college, workplace and healthcare settings	Alcohol and drugs	Fifty-two studies	No	Mewton et al. (28) identified three workplace-based reviews of alcohol or drug interventions. Contrasting conclusions were identified between the three reviews, with one systematic review of high-quality studies focusing on mandatory alcohol and drug testing noting some evidence of short-term reductions in injury, and some evidence of long-term effects. Comparatively, the review conducted by Lee et al. (18) found no support for the introduction of random alcohol testing in male-dominated industries A review by Webb et al. (66) identified that peer-based interventions, health promotion and interventions based on psychosocial skills training were found to be effective in reducing behaviors related to alcohol use. This overview of reviews noted a lack of literature examining universal prevention approaches within the workplace conducted since 2006 and further highlights the differences in effectiveness of intervention	4/7
Osilla et al. (29)	Wellness programs conducted in worksites	Alcohol	Thirty-three studies (three were relevant)	No	Using an RCT design, three studies assessed alcohol use frequency. Two studies reported reductions in alcohol use, including decreased drinking on weekends, frequency of intoxication and fewer days of alcohol consumption per week. Both studies implemented motivational interviewing-based interventions. One study, implementing a counseling-based treatment program found no impact, this may be due to the small sample size and a 3-year follow-up. This review highlighted the limited number of studies that evaluate treatment programs for early substance use problems as well as the limited number of RCTs with robust research design and sufficient follow up periods	5/7
Phillips et al. (21)	Occupational e-mental health interventions aimed at mental health, well-being, and alcohol misuse	Alcohol	Fifty studies (review)	Yes	Five studies that reviewed alcohol intake reported a range of effectiveness, across these studies it is unclear across as to how they define a reduction in alcohol consumption (I.e. improvements in binge drinking or drinking per weekday) Smal treatment effects were reported for two studies on alcohol intake, whilst two studies did not use comparable measures and reported improvements only for binge drinking and consumption reduction. The fifth study reported the opposite effect, a higher alcohol consumption in the intervention group. A meta-analysis of alcohol intake with two studies was completed. This indicated a small but non-significant effect on reducing alcohol intake, with g = 0.13 (95% CI −0.23–0.48, *P* = 0.488) This review illustrates the limits of a meta-analysis with small comparisons and the need for more studies measuring the effectiveness of e-health interventions on alcohol intake in the workplace	8/8
Schulte et al. (30)	Alcohol screening and brief interventions in workplaces, social service, and criminal justice settings	Alcohol	Nine (workplace) studies	No	Schulte et al. (30) reviewed studies that tested face-to-face alcohol screening and brief interventions (ASBI), web-based interventions alone or combined with the face-to-face approach. Eight of nine alcohol screening and brief interventions showed significant reductions in some of their primary outcomes such as alcohol intake or numbers of drinking days. Three out of four studies, which used web-based interventions reported some positive effects. In contrast to these studies, Araki et al. indicated that face-to-face educational interventions are more effective to increase the knowledge about and attitude toward drinking than a comparable email intervention Despite the effectiveness found above with the implementation of ASBI there is limited information regarding the effectiveness of ASBI in smaller sectors outside of manufacturing or construction. Schultz et al. (30) discusses the need for a comprehensive approach including healthy living policies and actions, as well as the structural and working environments that increase risky drinking	4/7
Watterson et al. (19)	Workplace-based interventions for reducing alcohol use in active-duty military personnel	Alcohol	Seven studies	No	All studies included active-duty military participants with an aim to implement interventions that reduce harmful or risky single-occasion drinking (RSOD). Participants were mostly males and outcome measures as well as follow-up periods varied widely. Three studies examined changes in individual or group attitudes and behaviors. Six studies demonstrated a significant effect on the outcomes of MI/BMI-based interventions, this is in line with success with similar age groups in the general population. Despite many of the studies being at risk of bias, they were able to demonstrate significant effects of the interventions when comparing baseline measures to early follow-up (up to 6months). For many studies this significance was not sustained for longer time follow-up periods. Watterson et al. (19) noted that effects at six months may still be important, indicating that interventions to reduce impacts in the short-term may prevent alcohol-related problems. Longer-term effects may reflect regression to the mean, seen in wider studies. The review noted the need for measurement of attitudinal change, particularly given the military drinking cultures. Individual-level measurements implemented across this review may not capture change in drinking culture and its role in changing problematic consumption	6/7
Weenink et al. (20)	Remediation and rehabilitation programmes for healthcare professionals with performance concerns.	Alcohol and drugs	Thirty eight studies, 19 were relevant	No	19 studies examined Physician Health Programmes (PHPs) aimed at treating healthcare professionals with substance use disorder and performance concerns. Many of the studies reported positive rehabilitation outcomes with high completion rate of 70–80% and 80-90% of participants either returning or remaining in practice. A review of 16 PHPs, over 5 years, 81% of physicians who completed treatment and resumed practice under supervision and monitoring remained abstinent. 19% of physicians relapsed (of whom 26% had a repeat positive test). Fourteen other studies reported abstinence rates between 56 and 86% for physicians, 60 and 94% for nurses, and 75% and 81% for healthcare professionals in general PHPs are unique in that they require participants to sign a formal, binding contract which includes intensive random alcohol/drug testing in combination with compliance monitoring and support. This may help explain why outcomes for PHPs are more effective than other forms of substance use treatment	5/7

AUDIT, Alcohol Use Disorders Identification Test; CDT, carbohydrate-deficient transferrin in serum; TLFB, Timeline Followback; EAP, Employee Assistance Program.

### Study selection

**Figure 1:**
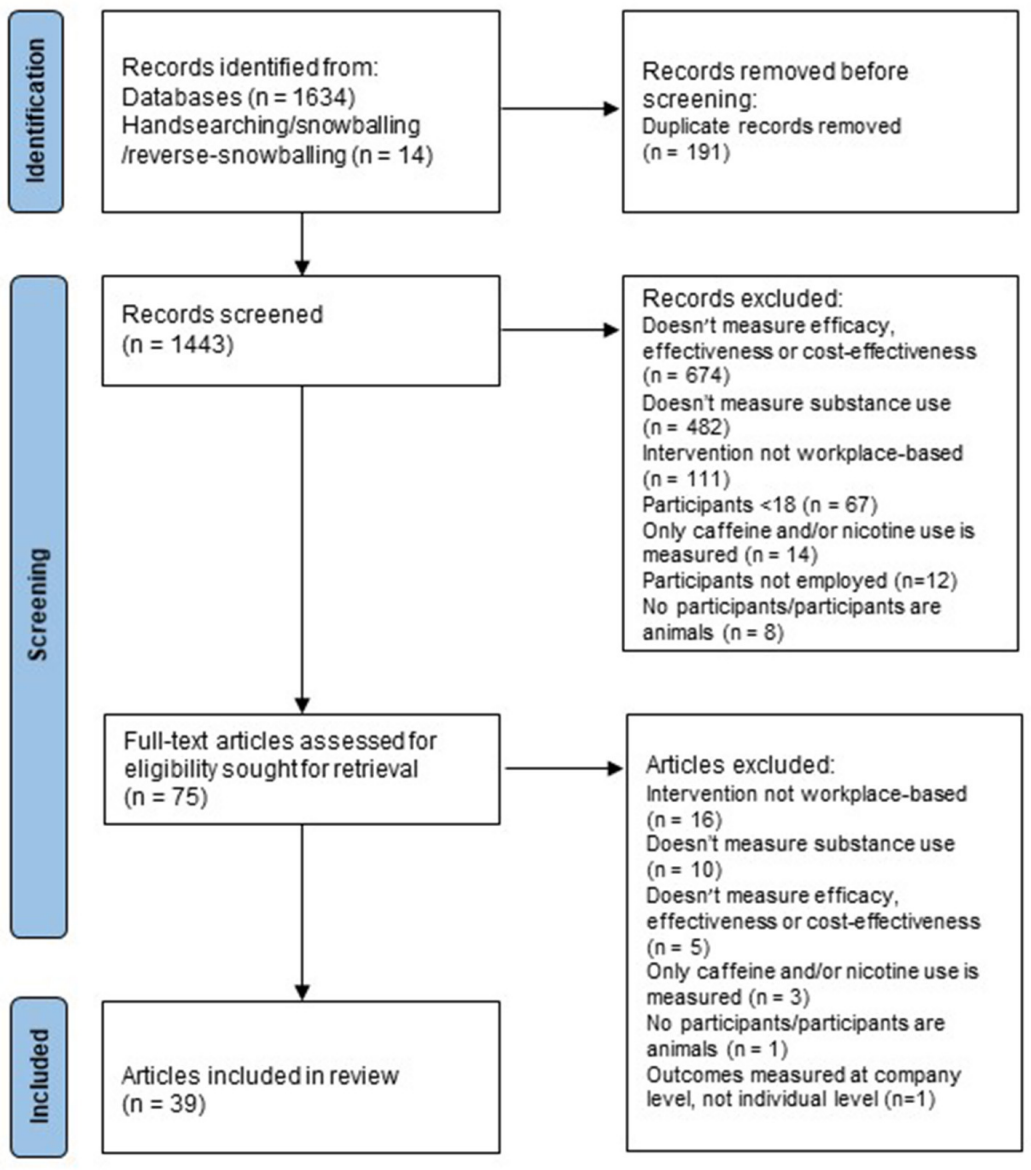
PRISMA flow diagram.

After removal of duplicates, the database searches identified 1,443 unique articles, 1,365 of which were excluded in title and abstract screening (see Figure 1). Of the 78 articles screened in full-text review, 39 met inclusion criteria; 28 were primary studies that evaluated a workplace-based intervention; 10 were systematic reviews, two of which conducted a meta-analysis (21, 22); and one was an overview of reviews (28).

### Study characteristics

Of 28 primary studies, 14 were randomized controlled trials (RCTs; 5 cluster-randomized), seven were quasi-experimental, and six used single-group pre-/post-test designs. Three studies conducted an economic evaluation (39, 45, 57). Most primary studies were conducted in the United States (US; *n* = 7) or United Kingdom (UK; *n* = 5); three in Sweden; two in each of the Netherlands, Japan and South Africa; and one in each of Norway, Spain, France, Russia, India and Australia. With regard to substance use outcomes, the overwhelming majority (27/28) solely reported alcohol use.

Baseline sample sizes ranged from 39 (34) to 5,362 (39). Follow-up rates ranged from 45.8% (39) to 100% (37), with most studies achieving follow-up rates of 60–80% at their final timepoint. Final follow-up ranged from 1-month to 3-years post-baseline. Studies recruited from a range of workforces, including white- and blue-collar workers across hospitality, retail, manufacturing, transportation, energy, health, education, law enforcement and military personnel, public administration, information technology, and financial services. Five studies did not report the type of workforce they recruited.

Most reviews (7/11) exclusively reviewed studies of alcohol use; three analyzed alcohol and other drug use (20, 25, 28), and one investigated only other drug use (23). The reviews include many primary studies conducted prior to 2010, and/or focused on specific workforces, intervention modalities and/or evaluation designs, so there was relatively little overlap in the primary studies included in this review and those analyzed in existing reviews.

### Effectiveness of workplace-based interventions

For all study information, including sample size, workforce characteristics, follow-up timepoints, and intervention characteristics, see Table 1. For all review characteristics, see Table 2. All primary studies examined the effectiveness of interventions rather than their efficacy. Thirteen primary studies evaluated *universal* interventions delivered to the entire workforce; 15 were *targeted* interventions delivered only to employees who met a certain risk threshold. Risk thresholds in these targeted studies included hazardous or harmful drinking (*n* = 10), cardiovascular disease (CVD) risk (*n* = 1), and having accessed an Employee Assistance Program (EAP) for mental health or alcohol and/or other drug use (*n* = 3). Of the 10 interventions delivered to workers who drank at hazardous or harmful levels, three excluded participants if they met criteria for alcohol dependence (41, 43, 49). Broad health promotion interventions were most common (*n* = 9), followed by brief interventions (BIs; *n* = 7), psychosocial interventions (*n* = 7), e-health interventions (*n* = 4), EAPs (*n* = 3), drug testing (*n* = 2), and stepped care (*n* = 1).

#### Broad health promotion interventions

There was evidence from both existing reviews and primary studies that universal broad health promotion interventions are associated with a reduction in alcohol use. Nine primary studies evaluated workplace-based broad health promotion interventions which addressed alcohol use amongst other health behaviors such as diet, exercise, stress, sleep, and smoking. Seven were universal interventions; one targeted at-risk drinkers (67); and one targeted employees at risk of developing CVD (46).

##### Reviews of health promotion interventions

Broad health promotion interventions are not focused on a single health domain or behavior, but address multiple domains/behaviors. Evidence from reviews was mixed. In their overview of reviews, Mewton et al. (28) identified one review that found evidence for health promotion interventions reducing substance use, but Mewton et al. (28) rated this review as low quality. In their systematic review, Osilla et al. (29) identified that two of three RCTs of worksite wellness interventions found an association between the intervention and a reduction in alcohol use. However, a recent meta-analysis of individual Dutch participant data (*n* = 44,007) found no impact of broad health promotion programs on units of alcohol consumed per week (22).

##### Primary studies of universal health promotion interventions

Of the seven universal interventions, five found modest but significant reductions in alcohol use following the intervention (39, 47, 48, 53, 54), whereas two did not (33, 58).

Three studies conducted single-group pre/post evaluations of broad health promotion interventions. Goetzel et al. (39) offered a multi-component health promotion intervention comprised of a health risk assessment, online resources, and phone-based health coaching to employees of small US businesses. At 1-year follow-up, a significantly smaller proportion of participants reported high alcohol use (≥15 drinks/week for men or ≥8 drinks/week for women) compared to baseline. LeCheminant et al. (48) evaluated a wellness intervention for US public school district employees, consisting of a health risk assessment, feedback, and educational exercises. Employees who reported using any alcohol at baseline consumed significantly fewer alcoholic drinks per day at 1-year and 2-year follow-up. Schouw et al. (53) similarly evaluated the impact of an intervention consisting of an Health Risk Assessment, feedback, education, and workplace-based resources such as healthy meals and exercise classes offered to South African power plant workers. At 2-year follow-up, a significantly smaller proportion were classified as harmful drinkers compared to baseline.

Of the four evaluations of universal interventions that included control groups, only one also analyzed within-group changes from baseline (i.e., pre-intervention) to follow-up. Findings relating to between-group differences were mixed.

Addley et al. (33) offered Northern Irish public service employees a comprehensive health risk assessment, followed by health and well-being education sessions and online modules and resources. They had two comparison groups: assessment-only, and no intervention. The authors found no significant within-group changes in alcohol use at 12-month follow-up, and no significant differences between the groups. Spicer and Miller (54) evaluated a modified form of the PREVENT health promotion intervention, originally designed for the US Navy, with young (aged 18–29) US railroad workers. PREVENT offered participants 2 days of group-based workshops on interpersonal issues, suicide, stress, smoking and alcohol use. Compared to assessment-only controls, participants offered PREVENT consumed significantly fewer alcoholic drinks in the past 30 days at follow-up (average time between baseline and follow-up assessment was 10.6 months for controls and 8.7 for treatment participants). Kuehl et al. (47) evaluated a safety and health improvement intervention with US law enforcement officers, which consisted of 12 half-hour team-based scripted sessions over 6-months, which addressed lifestyle behaviors such as exercise, diet, and sleep. Compared to a screening-only control group in a difference-in-difference analysis, participants randomized to receive the SHIELD intervention reported a significantly greater reduction in alcohol use from baseline to 12-month follow-up, but not from baseline to 6- or 24-month follow-up. Wierenga et al. (58) implemented the broadest health promotion intervention in Dutch university and hospital workplaces, which included informational posters, free fruit, and/or peer group counseling around work-related issues. The authors did not find a significant difference between intervention and control groups in glasses of alcohol consumed per week at follow-up, most likely because no intervention activities addressed alcohol use.

##### Primary studies of targeted health promotion interventions

Sieck and Heirich (67) evaluated the impact of a workplace wellness counseling intervention, but were unable to perform within- or between-group inferential statistics due to their small sample size. Kouwenhoven-Pasmooij et al. (46) screened Dutch public service (military, police and hospital) employees for CVD risk, and offered the treatment group a multicomponent lifestyle change intervention. At baseline, 65.4 and 86.6% of their sample did not meet the Dutch physical activity and diet guidelines, respectively; but only 11.8% exceeded the Dutch guidelines for alcohol consumption. At 12-month follow-up, participants offered the intervention reported a significant reduction in excessive alcohol use. However, the screening-only control group also reported a significant and comparable reduction in excessive alcohol use.

#### Brief interventions

There was evidence from reviews and primary studies that targeted BIs are associated with a reduction in alcohol use. However, evidence for the superiority of BIs over screening alone was mixed.

##### Reviews of brief interventions

In their systematic review, Kolar and von Treuer (26) concluded that there was no evidence for the effectiveness of workplace-based BIs for alcohol use. However, two other systematic reviews found evidence for the effectiveness of BIs for alcohol use in male-dominated workplaces (27) and for the effectiveness of workplace-based screening and BI (30).

##### Primary studies of universal brief interventions

Hagger et al. (68) conducted the only evaluation of a BI for all employees who consumed alcohol, not only those who met a certain alcohol use risk threshold. The BI consisted of alcohol use screening, followed by a task to set a goal of keeping alcohol use within World Health Organization guidelines. Participants randomized to receive the BI reported consuming significantly fewer weekly units of alcohol on average at 1-month follow-up than at baseline, while assessment-only controls did not significantly change their alcohol use. Compared to assessment alone, the BI group consumed significantly fewer weekly units of alcohol on average at 1-month follow-up.

##### Primary studies of targeted brief interventions

The remaining six BIs were offered only to participants who screened as hazardous or harmful drinkers, five of which compared the BI to a control condition. Joseph et al. (34) screened Indian hospital workers (manual laborers) for harmful drinking, and offered a feedback and advice BI to those who screened as moderate- to high-risk. At 4-month follow-up, participants reported significant reductions in alcohol use, desire to drink, and alcohol-related problems, compared to baseline. However, this finding should be interpreted with caution as the authors recruited a small sample (*n* = 39) and did not have a comparison group. Hermansson et al. (42) evaluated two forms of an alcohol intervention in Swedish transport workers against a screening-only control: one limited, one comprehensive. The limited intervention was a BI consisting of screening, feedback and advice; the comprehensive intervention offered this BI in addition to another assessment and keeping a drinking diary for 4 weeks. Regardless of group assignment, significantly fewer participants screened as harmful drinkers at 12-month follow-up compared to baseline. Similarly, Watson et al. (57) evaluated the impact of a BI consisting of screening and feedback on UK public service workers' alcohol use against screening-only, and found that regardless of group allocation, overall participants reported significantly lower AUDIT scores at 6-month follow-up. Ito et al. (43) evaluated two different BI interventions for harmful (but not dependent) drinking against an information-only control group in (predominantly) blue-collar employees of large Japanese companies. Both intervention groups received two 15-min BI sessions consisting of feedback, information about the risks of harmful alcohol consumption, goal-setting and coping strategies, and one additionally kept a drinking diary for 3 months. Both BI groups significantly reduced their average weekly drinks and binge drinking episodes and increased alcohol-free days at 3- and 12-month follow-up. Addley et al. (33) found that a limited (brief) multicomponent lifestyle intervention was associated with a significant reduction in the proportion of participants reporting excessive alcohol use at 12-month follow-up compared to baseline.

Three studies found that screening alone (42, 57) or screening and information about the negative consequences of heavy alcohol consumption (43) were also associated with significant reductions in alcohol use at follow-up, and were comparable to the BI. Three studies found no benefit of a more comprehensive intervention following a BI; Ito et al. (43) and Hermansson et al. (42) found no additional benefit of keeping a drinking diary for several weeks, and Addley et al. (33) found no additional benefit of seven health coaching sessions, in addition to a BI. Michaud et al. (49) recruited French employees attending required occupational medicine appointments who screened positive for hazardous drinking. Compared to controls who were randomized to receive a self-help booklet following screening, the BI group reported a significantly lower mean score on the Alcohol Use Disorders Identification Test (AUDIT), and significantly larger reductions in both alcohol use and AUDIT scores at 12-month follow-up. However, equivalent proportions of participants in the control and BI groups reduced their AUDIT score below the harmful threshold. The authors did not conduct within-group analyses.

#### Psychosocial interventions

Evidence for the effectiveness of psychosocial interventions was mixed. All three primary studies that found a psychosocial intervention was associated with reduced alcohol use evaluated modified forms of the “Team Awareness” intervention (59). However, evidence for the superiority of Team Awareness over control interventions was mixed.

##### Reviews of psychosocial interventions

Four reviews evaluated psychosocial interventions, which consisted of various combinations of education, problem-solving, role-playing and reinforcement exercises. Lee et al. (27) concluded that there is evidence to support the use of workplace peer-based psychosocial interventions that target alcohol use attitudes to prevent workplace injuries. They did not, however, find evidence for their effectiveness on alcohol use outcomes. In their overview of reviews, Mewton et al. (28) identified one review that included psychosocial skills-training interventions, which found them to be effective in reducing alcohol use. Kolar and von Treuer (26) found mixed evidence for the Team Awareness psychosocial intervention (59), and argued that it should be tailored to each workplace to be effective. Burnhams et al. (25) did not differentiate psychosocial interventions from other modalities in their analysis.

##### Primary studies of universal psychosocial interventions

Six out of seven psychosocial interventions were universal, three of which found some evidence of effectiveness in reducing alcohol use. Two studies evaluated an educational harm minimization intervention in Swedish workplaces, one short (2 × 45-min lectures) with finance/insurance salespeople (55), the other longer (2 x half-day sessions) with public service workers (56). Neither evaluation found that the intervention was associated with a reduction in alcohol use or AUDIT scores. Pidd et al. (50) evaluated a multi-component prevention, early intervention and treatment program for alcohol harm reduction in male-dominated Australian manufacturing worksites, and found no overall reduction in AUDIT scores at 12- or 24-month follow-ups. Reynolds and Bennett (51) evaluated a shortened form of the “Team Awareness” intervention with US small business employees. The original Team Awareness intervention offered skills-based training for referring peers to support for substance use, team building, and management of stress, as well as information about participants' employer's substance use policy (59). The authors found that participants reported drinking significantly less frequently, and on significantly fewer days out of the past month, at 6-month follow-up. The two remaining studies did not analyse within-group changes from baseline to follow-up (36, 38).

Three studies found that universal psychosocial interventions did not have a significant impact on alcohol use, and were comparable to assessment-only controls (50, 55, 56). In contrast, three studies reported some benefits of universal psychosocial interventions for some alcohol-related outcomes. Reynolds and Bennett (51) compared Team Awareness to a broad educational health promotion program (both delivered in one 4-h session), and screening-only control. Both intervention conditions were associated with comparable reductions in alcohol use, and were superior to screening alone. Broome and Bennett (36) delivered Team Awareness in three 2-h sessions to young US restaurant workers and assessed heavy drinking outcomes against a screening-only control group. The authors did not find a significant difference between Team Awareness and control participants in heavy drinking, but Team Awareness participants reported a significantly larger reduction in *recurrent* heavy drinking (heavy drinking on 5 or more days/month) at 6- but not 12-month follow-up, compared to controls. Team Awareness participants also reported significantly fewer alcohol-related problems at 12-month follow-up compared to controls. Burnhams et al. (38) delivered Team Awareness in eight 1-h sessions to South African public service employees, and compared them to those randomized to receive a 1-h general wellness talk. They found a significant group by time interaction, as Team Awareness participants reported a reduction, and control participants an increase, in binge drinking days in the past month from baseline to 3-month follow-up.

##### Primary studies of targeted psychosocial interventions

Harada et al. (41) conducted the only evaluation of a targeted psychosocial intervention for high-risk (but not dependent) drinkers in white-collar Japanese workplaces. They evaluated the “Haizen Alcoholism Prevention Program” (HAPPY) against a modified version (“HAPPY Plus”). The original HAPPY intervention offered three group sessions, consisting of alcohol use assessments, goal-setting, information about the health impacts of alcohol use, and keeping an alcohol-use diary. HAPPY Plus added group discussions, interactive education about health impacts to enhance participants' perception of alcohol use risks, and stress management. Both groups significantly reduced their average daily alcohol consumption and AUDIT scores from baseline to 3-month follow-up, but the groups were not significantly different at follow-up.

#### e-health interventions

Evidence from reviews suggested that e-health interventions may be effective for reducing alcohol use. However, the findings of four primary studies did not support this conclusion.

##### Reviews of e-health interventions

Two systematic reviews and one meta-analysis reported mixed evidence for the effectiveness of e-health interventions on alcohol use and binge drinking. Schulte et al. (30) reviewed BIs for alcohol use, including four web-based BIs, three of which found some evidence of effectiveness in terms of alcohol use reduction. Kolar and von Treuer (26) identified two evaluations of web-based interventions, both of which were found to be effective in reducing alcohol use. In their meta-analysis, Phillips et al. (21) identified two occupational e-health interventions for alcohol use, and found no significant impact of the interventions compared to passive control groups in a pooled analysis.

##### Primary studies of universal e-health interventions

Two studies evaluated broad health promotion programs that included web-based resources such as health-related articles and videos, and smoking and weight-loss behavior change programs (39) and online resources such as a personal trainer, monitoring resources and motivational messages (33). Goetzel et al. (39) found a significant reduction in harmful alcohol use at 1-year follow-up in a single-group pre/post study, whereas Addley et al. (33) found no impact of the intervention over screening alone or screening and feedback.

##### Primary studies of targeted e-health interventions

Two e-health interventions were targeted to high-risk drinkers. Brendryen et al. (35) evaluated a web-based self-help intervention for harmful drinking (using the Fast Alcohol Screening Test) consisting of feedback and 62 online sessions (with email reminders) over 6-months against a feedback and information control. Sessions were interactive and up to 10 min long, and involved quizzes and cognitive behavioral tasks. Participants could also opt-in to receive supportive text messages, but the authors did not report the number of participants that opted-in. Participants in both conditions significantly reduced their weekly alcohol use between baseline and 6-month follow-up, and were not significantly different at follow-up. Khadjesari et al. (45) screened employees in a large UK organization for a range of health behaviors and recruited those who screened as harmful drinkers on the AUDIT. Participants were randomized to receive feedback on all health behaviors plus a link to the multi-component web-based alcohol use intervention Down Your Drink (intervention), or feedback on all health behaviors *except* alcohol use (control). The authors did not analyses within-group changes from baseline to 3-month follow-up, and did not find a significant difference in past week alcohol use or AUDIT scores between the groups.

#### Employee assistance programs

Reviews and primary studies of EAPs often focused on workplace-level outcomes (e.g., accidents, insurance claims) and/or cross-sectional evaluation designs. There is no consistent evidence that EAPs are associated with a reduction in substance use.

##### Reviews of EAPs

Two reviews analyzed the effectiveness of EAPs, but the quality of evidence was poor. In their recent review of employer-led interventions for other drug use, Akanbi et al. (23) analyzed five studies that evaluated an EAP, two of which were cross-sectional. The other three studies assessed the effectiveness of an EAP on reducing workplace accidents or worker compensation claims but did not measure drug use. Weenink et al. (20) reviewed the effectiveness of mandated rehabilitation programs for clinicians whose work performance had been affected by substance use. Although completion rates were high (80–90%) and most clinicians returned to work following treatment, their analysis primarily focused on work-related outcomes, not alcohol and/or other drug use.

##### Primary studies of targeted EAPs

As only employees who seek help receive an intervention, all four EAP interventions were targeted. Only one study analyzed within-group changes in substance use from baseline to follow-up. Richmond et al. (52) compared risky alcohol use in US state government employees who had accessed their EAP to comparison participants who matched the EAP group on key characteristics such as demographic and employment characteristics. The EAP group did not report significantly different AUDIT scores, and were not significantly different from controls, at follow-up (range: 2- to 12-months).

Sieck and Heirich (67) evaluated the impact of a health promotion and substance use prevention intervention in conjunction with an EAP, but were unable to perform inferential analyses due to their small sample size. Jones et al. (44) recruited UK reserve forces personnel who had returned from deployment and accessed military-provided mental health treatment. The authors compared substance use outcomes in personnel whose mental health condition was attributable to their military service (treatment group) to those with non-attributable mental health issues (control group). Treatment group participants reported significantly higher AUDIT scores at baseline and follow-up compared to controls. Burgess et al. (37) evaluated the impact of an EAP at a Russian manufacturing worksite against a non-equivalent industrial worksite. At the intervention worksite, participants who self-identified or were identified by their employer as requiring an alcohol-use intervention were referred to the EAP. The authors found a significant condition by time interaction, whereby EAP participants significantly reduced their AUDIT scores from baseline to 90-day follow-up, but controls did not. However, EAP participants reported a substantially higher mean AUDIT score at baseline (13.79) than controls (3.59).

#### Substance use testing

There was a focus on workplace-level outcomes (e.g., accidents, insurance claims) and/or cross-sectional evaluation designs in the substance use testing literature. There was little evidence to support workplace-based testing.

##### Reviews of substance use testing

Four reviews analyzed the effectiveness of workplace substance use testing, but these analyses tended to use poor quality studies and/or focus on workplace-level outcomes such as accidents and injuries. Lee et al. (27) concluded that workplace alcohol testing was not effective to reduce alcohol use in male-dominated (i.e., on average >70% male at the industry level) workplaces such as construction, mining, transport and manufacturing. In contrast, Kolar and von Treuer (26) argued that random alcohol and other drug testing is highly effective; however, only one of the three studies they reviewed reported that testing reduced substance use at the individual level. In their overview of reviews, Mewton, Visontay (28) found mixed evidence for the effectiveness of workplace substance use testing; the highest quality review in their analysis that found testing to be effective reported positive impacts on workplace injuries, not substance use *per se*. Akanbi et al. (23) reviewed 11 evaluations of workplace drug testing programs, five of which measured substance use. Two of these studies found an association between testing and lower rates of employee substance use, but both were cross-sectional designs and did not assess change over time.

##### Primary studies of universal substance use testing

Two primary studies evaluated the impact of universal workplace substance use testing. Sieck and Heirich (67) reported that an increase in random drug testing (RDT) at a manufacturing worksite did not significantly impact the proportion of employees reporting at-risk alcohol use. However, they measured use at each time point using anonymous surveys that were not linked to individuals, so it is unclear to what extent the baseline and follow-up survey respondents overlap. Gómez-Recasens et al. (40) introduced universal alcohol and other drug monitoring (self-report and urine screening) at multiple Spanish industrial worksites and evaluated its effectiveness in a single group pre-/post-intervention analysis. The proportion of employees reporting risky alcohol use significantly declined from baseline to 1-year follow-up, and from 1-year to 2-year follow-up (reduction maintained at 3-year follow-up). The proportion of employees reporting any other drug use did not significantly change from baseline to 1-year follow-up or 2- to 3-year follow-up, and modestly but significantly increased from 1-year to 2-year follow-up. However, Gómez-Recasens et al. (40) implemented universal substance use monitoring as part of a stepped-care intervention, so these effects may be attributable to the other interventions offered to employees (described below).

#### Stepped-care

Only one article described the effectiveness of a stepped-care intervention. Gómez-Recasens et al. (40) introduced universal alcohol and other drug monitoring in Spanish industrial worksites and referred employees who screened positive for risky alcohol use, or any other drug use to further intervention. Depending on their use severity, employees were offered a brief intervention or referral to specialist substance use treatment. As described above, the stepped-care model was associated with a significant reduction in risky alcohol use, although it is unclear which component/s this effect can be attributed to.

### Cost-effectiveness

Only three studies conducted an economic evaluation. Goetzel et al. (39) evaluated the return-on-investment of a universal intervention addressing ten health risk behaviors, including poor diet, insufficient exercise, smoking and harmful alcohol use. The authors did not use a control group, instead comparing predicted and actual changes in employee health risk scores from baseline to 1-year follow-up and the associated financial savings from reductions in health risks through use of an existing return on investment model. The statistically significant reductions in seven risk factors (obesity, poor eating habits, poor physical activity, tobacco use, high alcohol consumption, high stress and depression) and non-significant improvements in high blood pressure, high total cholesterol and high blood glucose were estimated to result in medical and productivity cost savings and a total return-on-investment of $2.03 for every $1 spent on the intervention. The contribution of reduced alcohol consumption to the overall financial benefit is unclear. Khadjesari et al. (45) compared outcomes including preference based utility values (EQ-5D) and the cost of self-reported health care resource use and number of sick days between employees receiving feedback on alcohol in addition to other health risks compared to employees only receiving feedback on health risks. At 3-month follow-up there were no significant differences in AUDIT scores, utility values or costs. Watson et al. (57) conducted a pilot trial of a BI delivered by an occupational health nurse which resulted in a mean savings of £344.5 per intervention participant, and a very small average benefit of 0.008 quality adjusted life years (QALYs) compared to a screening only control group.

### Workforce characteristics associated with intervention outcomes

As discussed, the primary studies reviewed were conducted across a range of industries. No studies allowed for a direct comparison between industries, and only one study analyzed the moderating effect of worker roles (42). A limited range of other workforce characteristics were examined across a small number of studies, including age, sex, role characteristics, substance use severity, attitudes, and socioeconomic status.

#### Age

Age was associated with intervention outcomes in two of three primary studies that analyzed it as a moderator. Hermansson et al. (42) and Michaud et al. (49) found that age was significantly associated with change in AUDIT score from baseline to follow-up, such that younger participants were more likely to report a score reduction at follow-up. Hermansson et al. (42) analyzed the relationship between age and difference in AUDIT score from baseline to follow-up independent of other variables, whereas Michaud, Kunz (49) controlled for AUDIT score at baseline and group allocation. Kuehl et al. (47) did not find that age moderated intervention outcomes.

#### Sex

There were mixed findings for the impact of sex on intervention outcomes. Broome and Bennett (36) found that male participants reported significantly more recurring heavy drinking (5 or more days in the past 30 of consuming 5 or more drinks) at baseline compared to female participants but did not examine the impact of sex on changes in alcohol use from baseline to follow-up. Three studies found that sex did not moderate changes in substance use from 12 to 24 months (42, 47, 50), whereas two studies reported greater reductions in alcohol use amongst male participants compared to female participants. Michaud et al. (49) found that baseline AUDIT scores were significantly higher amongst male participants compared to female participants, and that only males in the intervention group significantly reduced their alcohol use at follow-up compared to (male) controls. Similarly, Sieck and Heirich (67) found that women reported significantly higher perceived risks of alcohol use and significantly lower alcohol consumption at baseline compared to men, and that men reported significantly larger reductions in their alcohol use at follow-up compared to women.

#### Role characteristics

Hermansson et al. (42) did not find that the timing of shiftwork (day vs. night) or whether participants were employed in a manual or non-manual role impacted intervention outcomes.

#### Substance use severity

Evidence for the moderating effect of substance use severity on intervention outcomes was weak. Tinghög and Tinghög (56) did not find an overall intervention effect, but found that amongst the heaviest drinkers (≥6.4 g/day), only those allocated to receive the intervention significantly reduced their alcohol use at follow-up relative to heavy-drinking controls. There was no effect of group allocation amongst the lightest drinkers (≤4.14 g/day). However, Tinghög (55) did not find the same effect of heavy drinking. Richmond et al. (52) found that regardless of treatment condition, baseline AUDIT scores predicted scores at follow-up, whereas Michaud et al. (49) found no association between baseline AUDIT scores and intervention outcomes.

#### Attitudes

Reynolds and Bennett (51) found that changes in attitudes toward help-seeking predicted whether employees' sought counseling for alcohol use but did not predict alcohol use outcomes.

#### Socioeconomic status

Coenen et al. (22) found that socioeconomic status did not predict compliance with workplace-based interventions or their effectiveness for reducing alcohol use.

### Barriers and facilitators

No primary studies or reviews directly examined the barriers to and/or facilitators for implementing workplace-based interventions. Lee et al. (27) and Schulte et al. (30) noted in their reviews that male employees may be more likely to experience issues with alcohol use, but less likely to seek help for them, compared to female employees. Schulte et al. (30) and Watson et al. (57) identified concerns around confidentiality and stigma as barriers to workplace-based alcohol and other drug screening, as employees may be concerned about consequences for their career if they disclose a substance use issue.

Tailoring and ease of implementation/engagement emerged as facilitators. In their review, Kolar and von Treuer (26) argued that interventions likely need to be tailored to each workplace to be effective. Echoing this conclusion, Wierenga et al. (58) found that participants reported a preference for personalized interventions. Wierenga et al. (58) also found that employees preferred interventions that were simple, easy and cheap to implement, and those that were easily accessible and did not incur significant time costs (e.g., interventions integrated into existing meetings).

### Study quality

See Table 1 for primary study quality ratings. Half (14/28) of the included studies were rated “poor” quality according to Korokakis et al.'s (24) modified version of the Downs and Black Quality Index (1998), six were “fair” quality, and eight were “good” quality. No primary studies were rated “excellent”. In general, these scores reflect low scores for internal validity (i.e., high risk of bias) and underpowered studies.

See Table 2 for review quality ratings. Of the 11 reviews, two included a meta-analysis; one scored 7/8 (22) and the other scored 8/8 (21) on the NIH quality assessment tool (31). Of the nine reviews rated out of 7, one scored 7 (27), two scored 6, three scored 5, and three scored 4.

The quality scores for the three included economic evaluations ranged from fair (7.5/10 points) for Khadjesari et al. (45) to poor for Goetzel et al. (39) (5.5/10 points) and Watsonet al. (57) (4/10 points). Watson et al. (57) did not identify the perspective of the analysis or identify the sources of unit costs. Goetzel et al. (39) did not evaluate clinical or quality of life outcomes and used an existing economic model without details of the unit costs included. Khadjesari et al. (45) included healthcare resource use and sick leave costs reported by participants but did not include the cost of the intervention.

## Discussion

Given considerable changes in the nature of the workplace and working arrangements, particularly in the last decade, this review provides a timely synthesis of the international evidence regarding the efficacy, effectiveness and cost-effectiveness of workplace-based interventions for the prevention and treatment of problematic substance use. Over the last decade, interventions have been tested across a broad range of workforces, including public servants, young restaurant staff, and manufacturing workers. A range of reviews of workplace-based interventions have also been published with differing focus points including occupational medicine, BIs, and male-dominated workplaces. The vast majority of research focused on alcohol use.

Heterogeneity between studies regarding workforce (e.g., white or blue collar; industry; organization size), intervention design (e.g., universal or targeted; focused on substance use or addressed a range of health behaviors; single- or multi-component) and evaluation approach (e.g., study design; measures used; analysis of within- or between-groups differences) limited the degree to which the data could be synthesized to draw robust conclusions. In addition, most primary studies were rated as poor quality. Although there was insufficient (and mixed) evidence to determine the overall effectiveness and cost-effectiveness of workplace-based interventions for problematic substance use, there was some promising evidence to support workplace-based universal broad health promotion interventions, targeted BIs, and universal screening.

Consistent with earlier reviews (28, 29), this review found evidence that universal broad health promotion interventions are associated with a reduction in substance use. Most (5/7) universal health promotion interventions were associated with a reduction in alcohol use, and two studies of a psychosocial alcohol-focused intervention (Team Awareness) found it to be equivalent to a broad health and wellness intervention on at least one outcome measure (38, 51). However, the quality of these studies tended to be poor; only 4/7 studies included a control group. Workplace wellness programs are increasingly common, particularly in larger US organizations (69, 70). However, these programs do not consistently address substance use beyond smoking (71). There is some evidence that workplace wellness interventions can improve a range of health behaviors (72), but more high-quality evidence is needed to determine their effectiveness for substance use.

All but one of the primary studies that evaluated a BI examined targeted interventions, and all found some evidence of effectiveness in reducing alcohol use. Moreover, four evaluations of more intensive intervention modalities found that control BIs were associated with equivalent reductions in alcohol use as compared to intensive interventions (35, 42, 43, 46). However, there was mixed evidence with regard to the superiority of BIs over screening alone, as 3/5 BI evaluations that included a control group found no significant differences between intervention and control at follow-up. Inconsistent evidence for the effectiveness of BIs for alcohol use could be due to heterogeneity in intervention approach (e.g., motivational vs. informational) and intensity (e.g., 5 vs. 60+ minutes), comparison groups (e.g., screening-only vs. screening and information/feedback), severity of substance use at baseline (e.g., low- vs. high-risk), or lack of consideration for the cultural context in which the BI was applied (73).

There was comparatively weak evidence to support the use of workplace-based psychosocial or e-health interventions. The most promising evidence from primary studies of psychosocial interventions was for Team Awareness (59), but 2/3 studies found that a general health intervention produced comparable outcomes. Of the four reviews that assessed psychosocial interventions, only Mewton et al. (28) found evidence of their effectiveness, specifically for skills-based interventions. Consistent with three previous reviews (21, 26, 30), no significant differences were found between intervention and control groups in three of four evaluations of e-health interventions (35, 45) or those with web-based components (33). Phillips et al. (21) note that attrition rates are often high in e-health interventions; indeed, only 3% of Khadjesari et al.'s (45) treatment group registered for the intervention website (Down Your Drink), and only five participants (12% of the treatment group) completed all 62 web sessions in Brendryen et al.'s (35) study. In addition, Wierenga et al. (58) reported that participants did not engage with the online components of their multicomponent intervention – they did not visit the website, or read emails about the program. Engagement should be a key consideration for implementation of e-health interventions, as they provide a range of benefits over face-to-face intervention modalities, such as providing accessibility and anonymity to participants who would otherwise be unable or unwilling to seek help, and reducing healthcare costs (74).

Unfortunately at this time, the quality of primary studies and those included in reviews were particularly poor, and cannot be used to draw robust conclusions about the effectiveness of Employee Assistance Programs (EAPs) or workplace substance use testing. In all four EAP evaluations, participants self-selected into treatment, follow-up periods varied within and between EAP and comparison groups, the treatment offered to EAP participants was unclear, and comparison groups were systematically different from EAP groups. The lack of evidence on the effectiveness of EAPs for substance use may be explained by a focus on workplace-level outcomes such as absenteeism in EAP evaluations, driven by organizations' need to demonstrate return-on-investment (75). Similarly, evaluations of workplace substance use testing tend to focus on workplace-level safety outcomes and/or use cross-sectional designs, limiting their utility for determining the effect of testing on employees' substance use (76).

Six primary studies (35, 42, 43, 45, 46, 49, 57) and two reviews (27, 28) found that screening alone was associated with a significant reduction in substance use, suggesting that screening is itself a substance use intervention. Indeed, a meta-analysis of the impact of screening in the absence of any subsequent intervention found that completing an assessment was associated with a significant reduction in alcohol use (77). In settings such as primary healthcare, screening is used in conjunction with a BI and/or specialist referral to safely guide people using substances at harmful levels into treatment (78), an approach that has been underutilized in the workplace (79). It is crucial that workplaces use effective screening measures to identify at-risk employees to facilitate early intervention (80).

Few primary studies analyzed workforce characteristics as moderators of treatment outcomes. There was mixed evidence for sex, age and substance use severity moderating intervention outcomes; however, it appears that participants reporting heavier substance use at baseline (who tend to be young and/or male) are more likely to report a reduction at follow-up.

The workplace is a complex intervention context as employers must balance their duty of care to employees (81) against safety concerns resulting from substance-induced impairment (76, 82, 83). It is therefore important to consider the barriers and facilitators to implementing a substance use intervention in the workplace alongside evaluating effectiveness and cost-effectiveness. Unfortunately, few primary studies or reviews have considered implementation factors. However, the overwhelming focus on alcohol use outcomes (27/28 primary studies) suggests that addressing other drug use in the workplace is more challenging. Lack of engagement with e-health interventions, heavier use and reluctance to seek help amongst male employees (compared to female employees), and concerns around confidentiality, emerged as barriers. Intervention tailoring, and ease of implementation and engagement were identified as facilitators in the workplace. More evidence on workplace-specific implementation factors is needed, ideally through process evaluations.

The scarcity of high-quality economic evaluations of workplace interventions to reduce substance use may be due to the limited time-horizons, difficulty collecting cost and outcome data alongside trials or the complexity of the workplace as a setting for intervention. Businesses may also rely on previous literature suggesting health promotion programs result in lower absenteeism and health care costs as sufficient evidence for implementation (84), despite more recent research suggesting that study quality may influence findings (85).

Primary studies and reviews conducted over the past decade have revealed some promising evidence for universal health promotion interventions, targeted brief interventions, and universal substance use screening. The sparse evidence around factors influencing implementation suggests that preserving confidentiality (and assuring employees that their disclosures will not be provided to their employer), tailoring interventions to each workplace, and making interventions easy to implement and engage with may assist implementation. There is a need for future studies to address implementation, as well as use of substances other than alcohol.

## Data availability statement

The original contributions presented in the study are included in the article/Supplementary material, further inquiries can be directed to the corresponding author/s.

## Author contributions

AM, MA, and JS developed the database search strategy, screened articles, extracted data from included articles, analyzed extracted data, and drafted and revised the manuscript. KD analyzed extracted data and summarized the findings of the included systematic reviews. KM revised the database search strategy and drafted and revised the manuscript. M-LC drafted and revised the economic analyses in the paper and reviewed the draft manuscript. AF and CM revised the database search strategy and reviewed the draft manuscript. FK-L, MS, LH, NP, and MT reviewed the draft manuscript. All authors contributed to the article and approved the submitted version.

## Funding

Funding for this review was provided by icare NSW. KM, CM, FK-L, EB, and MT received fellowship funding from the Australian National Health and Medical Research Council.

## Conflict of interest

The authors declare that the research was conducted in the absence of any commercial or financial relationships that could be construed as a potential conflict of interest.

## Publisher's note

All claims expressed in this article are solely those of the authors and do not necessarily represent those of their affiliated organizations, or those of the publisher, the editors and the reviewers. Any product that may be evaluated in this article, or claim that may be made by its manufacturer, is not guaranteed or endorsed by the publisher.
