# Supplementary material for: A systematic review of the efficacy, effectiveness and cost-effectiveness of workplace-based interventions for the prevention and treatment of problematic substance use
Source: Front Public Health. 2022 Nov 7;10:1051119. doi: 10.3389/fpubh.2022.1051119 (PMC9676969; doi:10.3389/fpubh.2022.1051119)
Supplement: Supplementary file 1 [file Data_Sheet_1.PDF]

PI(E)COS Worksheet and Search Strategy

1. *Define your question using PI(E)COS by identifying: Patient/Population/Problem; Intervention/Exposure, Comparison/Control Group and Outcome/s*

Patient/Population/Problem:

1. People aged 18 years or older (i.e. adults)
2. Currently employed

Intervention:
Workplace-based interventions

Interventions must meet the following criteria to be considered a workplace-based intervention:

1. Interventions must be sourced through, subsidised or provided by the employer (e.g potentially eligible interventions would be drug screening / testing, screening / assessment and referral, psychoeducation, group-based or individual psychosocial interventions, tele- or e-health, peer-support groups or self-help programs).

Comparison/Control:
Studies with or without a control group (e.g., usual care, active control, wait list control)

Outcomes of Relevance:
Substance use outcomes measured at the level of the individual (e.g. quantity and/or frequency of substance use measured via self-report of biological testing; severity of symptoms of substance use disorder measured by scores on self- or clinician-rated diagnostic questionnaires). Substance use outcomes at the organisational level will not be included in the review.

***2.** Type of question/problem: Treatment/Therapy/Intervention/Exposure*

Primary question:

What is the efficacy, effectiveness and cost-effectiveness of workplace-based interventions for the prevention and treatment of substance use?

Secondary questions:

(a) Which modalities of workplace-based interventions are effective for preventing and/or treating problematic substance use?

(b) How do characteristics of the workplace or the workforce impact on which types of interventions are most effective to prevent and/or treat problematic substance use?

(c) What are the challenges and difficulties in implementing interventions for different drug classes in the workplace?

3. *Types of studies/publication to include in the search*

**Study designs INCLUDED in the review**	**Study designs EXCLUDED from the review**
Primary research studies, including:<br>• Randomised controlled trials and other experimental studies<br>• Quasi-experimental studies (e.g. uncontrolled trials, pre-test/post-test designs)<br><br>Literature reviews, including:<br>• Systematic reviews<br>• Narrative reviews<br>• Meta-analyses<br>• Economic evaluations	Non-intervention/treatment studies, including:<br>• Observational studies (e.g. cross-sectional surveys, cohort and case studies)<br>• Qualitative studies<br>• Case report or series<br>• Protocol and conference papers<br>• Editorials or commentaries<br><br>Animal studies

4. *List the main topics and alternative terms from your PI(E)COS question that can be used for your search:*

Condition (alcohol or other drug related terms)

substance use / drug use / alcohol use / substance abuse / drug abuse / alcohol abuse / substance dependence/ drug dependence / alcohol dependence / substance use disorder / drug use disorder / alcohol use disorder / SUD / hazardous drinking / problem drinking / harmful drinking / risky drinking / drug testing

Subject heading

MeSH (MEDLINE/PubMed; Cochrane; CINAHL)

- substance-related disorders

Emtree (EMBASE)

- substance abuse
- drug abuse

APA's Thesaurus of Psychological Index Terms (PsychINFO)

- substance use disorder

Interventions (prevention or treatment related terms)

workplace / work-place / workforce / work-force / worksite / work-site / employee* / job* / employer* / vocation* / compan* / office / offices / factory / factories

AND

prevent* / program* / intervention* / train* / polic* / treatment* / service* / promotion* / education/ psychoeducation / peer-support* / peer support* / self-help* / self help*/support* / testing

Subject heading

MeSH (MEDLINE/PubMed; Cochrane; CINAHL)

- occupational health services

APA's Thesaurus of Psychological Index Terms (PsychINFO)

- employee assistance programs
- workplace intervention

Efficacy and effectiveness terms

RCT / randomi?ed controlled trial* / clinical trial*/ efficacy trial*/ efficacy evaluation*/ systematic review*/ meta analy*/ meta-analy*/ effectiveness evaluation*/ pragmatic trial*/ effectiveness trial*/ outcome evaluation*/ impact evaluation*/ objective-based evaluation*/ summative evaluation*

Subject headings

MeSH (MEDLINE/PubMed; Cochrane; CINAHL)

- treatment outcome

Emtree (EMBASE)

- treatment outcome

APA's Thesaurus of Psychological Index Terms (PsychINFO)

- treatment outcomes

Cost-effectiveness terms

cost benefit analys?s/ cost-benefit analys?s/ economic evaluation*/ costs and cost analys?s/ cost analys?s/ return on investment/ return to investment/ cost effectiveness analys?s/ cost-effectiveness analy?s/ cost utility analys?s/ cost effectiveness evaluation*/ cost-effectiveness evaluation*/ cost-utility analys?s

Subject headings

MeSH (MEDLINE/PubMed; Cochrane; CINAHL)

- cost-benefit analysis

Emtree (EMBASE)

- cost benefit analysis

5. Write out your search strategy (adapt for each database)

PsychINFO (via Ovid)

1. exp "Substance Use Disorder"/
2. ((use* or abuse* or misuse* or dependenc* or addict* or disorder* or problem* or hazard* or harm* or risk* or test*) adj4 (substance or drug* or alcohol* or amphetamine* or cannabis or marijuana or cocaine or inhalant* or hallucinogen* or phencyclidine or heroin or morphine or opioid* or stimulant* or sedative* or hypnotic* or anxiolytic* or benzodiazepine*)).mp.
3. SUD.mp.
4. **1 or 2 or 3**
5. ((organi?ation* or busines* or labor or labour or corporat* or occupation* or workplace or work-place or workforce or work-force or worksite or work-site or employee* or job* or employer* or vocation* or compan* or office or offices or factory or factories) adj4 (prevent* or program* or intervention* or train* or polic* or treatment* or service* or promotion* or education* or psychoeducation or peer-support* or peer support* or self-help* or self help* or support* or test*)).mp.
6. ("employee assistance program" or EAP or "critical incident stress debriefing" or CISD).mp.
7. workplace intervention/ or employee assistance programs/
8. **5 or 6 or 7**
9. treatment outcomes/
10. ("cost benefit analys?s" or "cost-benefit analys?s" or "economic evaluation*" or "cost and cost anals?s" or "cost analys?s" or "return on investment" or "return to investment" or "cost effectiveness analys?s" or "cost-effectiveness analys?s" or "cost utility analysis" or "cost-utility analysis" or "cost effectiveness evaluation*' or cost-effectiveness evaluation* or RCT or randomi?ed controlled trial* or clinical trial* or efficacy trial* or efficacy evaluation* or systematic review* or meta analy* or meta-analy*" or "effectiveness evaluation*" or "pragmatic trial*" or "effectiveness trial*" or "outcome evaluation*" or "impact evaluation*" or "objective-based evaluation*" or "summative evaluation*").tw.
11. **9 or 10**
12. **4 and 8 and 11**

<u>Medline (via Ovid)</u>

1. exp Substance-Related Disorders/
2. ((use* or abuse* or misuse* or dependenc* or addict* or disorder* or problem* or hazard* or harm* or risk* or test*) adj4 (substance or drug* or alcohol* or amphetamine* or cannabis or marijuana or cocaine or inhalant* or hallucinogen* or phencyclidine or heroin or morphine or opioid* or stimulant* or sedative* or hypnotic* or anxiolytic* or benzodiazepine*)).mp.
3. SUD.mp.
4. **1 or 2 or 3**
5. ((organi?ation* or busines* or labor or labour or corporat* or occupation* or workplace or work-place or workforce or work-force or worksite or work-site or employee* or job* or employer* or vocation* or compan* or office or offices or factory or factories) adj4 (prevent* or program* or intervention* or train* or polic* or treatment* or service* or promotion* or education* or psychoeducation or peer-support* or peer support* or self-help* or self help* or support* or test*))).mp.
6. ("employee assistance program" or EAP or "critical incident stress debriefing" or CISD).mp.
7. **5 or 6**
8. Treatment Outcome/
9. Cost-Benefit Analysis/
10. ("cost benefit analys?s" or "cost-benefit analys?s" or "economic evaluation*" or "cost and cost anals?s" or "cost analys?s" or "return on investment" or "return to investment" or "cost effectiveness analys?s" or "cost-effectiveness analys?s" or "cost utility analysis" or "cost-utility analysis" or "cost effectiveness evaluation*' or cost-effectiveness evaluation* or RCT or randomi?ed controlled trial* or clinical trial* or efficacy trial* or efficacy evaluation* or systematic review* or meta analy* or meta-analy*" or "effectiveness evaluation*" or "pragmatic trial*" or "effectiveness trial*" or "outcome evaluation*" or "impact evaluation*" or "objective-based evaluation*" or "summative evaluation*").tw.
11. **8 or 9 or 10**
12. **4 and 7 and 11**

EMBASE

1. exp substance abuse/
2. exp drug abuse/
3. ((use* or abuse* or misuse* or dependenc* or addict* or disorder* or problem* or hazard* or harm* or risk* or test*) adj4 (substance or drug* or alcohol* or amphetamine* or cannabis or marijuana or cocaine or inhalant* or hallucinogen* or phencyclidine or heroin or morphine or opioid* or stimulant* or sedative* or hypnotic* or anxiolytic* or benzodiazepine*)).mp.
4. SUD.mp.
5. **1 or 2 or 3 or 4**
6. ((organi?ation* or busines* or labor or labour or corporat* or occupation* or workplace or work-place or workforce or work-force or worksite or work-site or employee* or job* or employer* or vocation* or compan* or office or offices or factory or factories) adj4 (prevent* or program* or intervention* or train* or polic* or treatment* or service* or promotion* or education* or psychoeducation or peer-support* or peer support* or self-help* or self help* or support* or test*)).mp.
7. ("employee assistance program" or EAP or "critical incident stress debriefing" or CISD).mp.
8. **6 or 7**
9. treatment outcome/
10. cost benefit analysis/
11. ("cost benefit analys?s" or "cost-benefit analys?s" or "economic evaluation*" or "cost and cost anals?s" or "cost analys?s" or "return on investment" or "return to investment" or "cost effectiveness analys?s" or "cost-effectiveness analys?s" or "cost utility analysis" or "cost-utility analysis" or "cost effectiveness evaluation*' or cost-effectiveness evaluation* or RCT or randomi?ed controlled trial* or clinical trial* or efficacy trial* or efficacy evaluation* or systematic review* or meta analy* or meta-analy*" or "effectiveness evaluation*" or "pragmatic trial*" or "effectiveness trial*" or "outcome evaluation*" or "impact evaluation*" or "objective-based evaluation*" or "summative evaluation*").tw.
12. **9 or 10 or 11**
13. **5 and 8 and 12**

Scopus

(TITLE-ABS-KEY (((use* OR abuse* OR misuse* OR dependenc* OR addict* OR disorder* OR problem* OR hazard* OR harm* OR risk* or test*) W/4 (substance OR drug* OR alcohol* OR amphetamine* OR cannabis OR marijuana OR cocaine OR inhalant* OR hallucinogen* OR phencyclidine OR heroin OR morphine OR opioid* OR stimulant* OR sedative* OR hypnotic* OR anxiolytic* OR benzodiazepine*)) OR SUD))

AND

(TITLE-ABS-KEY (((organi?ation* OR busines* OR labor OR labour OR corporat* OR occupation* OR workplace OR work-place OR workforce OR work-force OR worksite OR work-site OR employee* OR job* OR employer* OR vocation* OR compan* OR office OR offices OR factory OR factories) W/4 (prevent* OR program* OR intervention* OR train* OR polic* OR treatment* OR service* OR promotion* OR education* OR psychoeducation OR peer-support* OR "peer support*" OR self-help* OR "self help*" OR support* or test*)) OR "employee assistance program" OR EAP OR "critical incident stress debriefing" OR CISD))

AND

(TITLE-ABS-KEY ("randomi?ed controlled trial*" OR "clinical trial*" OR "efficacy trial*" OR "efficacy evaluation*" OR "systematic review*" OR "meta analy*" OR "meta-analy*" OR "effectiveness evaluation*" OR "pragmatic trial*" OR "effectiveness trial*" OR "outcome evaluation*" OR "impact evaluation*" OR "objective-based evaluation*" OR "summative evaluation*" OR "cost benefit analys?s" OR "cost-benefit analys?s" OR "economic evaluation*" OR "costs and cost analys?s" OR "cost analys?s" OR "return on investment" OR "return to investment" OR "cost effectiveness analys?s" OR "cost-effectiveness analy?s" OR "cost utility analys?s" OR "cost effectiveness evaluation*" OR "cost-effectiveness evaluation*" OR "cost-utility analys?s" OR RCT))

Cochrane Database

1. exp substance-related disorders/
2. (((use* OR abuse* OR misuse* OR dependenc* OR addict* OR disorder* OR problem* OR hazard* OR harm* OR risk* OR test*) adj4 (substance OR drug* OR alcohol* OR amphetamine* OR cannabis OR marijuana OR cocaine OR inhalant* OR hallucinogen* OR phencyclidine OR heroin OR morphine OR opioid* OR stimulant* OR sedative* OR hypnotic* OR anxiolytic* OR benzodiazepine*)) OR SUD)
3. occupational health services/
4. (((organi?ation* OR busines* OR labor OR labour OR corporat* OR occupation* OR workplace OR work-place OR workforce OR work-force OR worksite OR work-site OR employee* OR job* OR employer* OR vocation* OR compan* OR office OR offices OR factory OR factories) adj4 (prevent* OR program* OR intervention* OR train* OR polic* OR treatment* OR service* OR promotion* OR education* OR psychoeducation OR peer-support* OR "peer support*" OR self-help* OR "self help*" OR support* OR test*)) OR "employee assistance program" OR EAP OR "critical incident stress debriefing" OR CISD)
5. Treatment outcome/
6. Cost-benefit analysis/
7. ("randomi?ed controlled trial*" OR "clinical trial*" OR "efficacy trial*" OR "efficacy evaluation*" OR "systematic review*" OR "meta analy*" OR "meta-analy*" OR "effectiveness evaluation*" OR "pragmatic trial*" OR "effectiveness trial*" OR "outcome evaluation*" OR "impact evaluation*" OR "objective-based evaluation*" OR "summative evaluation*" OR "cost benefit analys?s" OR "cost-benefit analys?s" OR "economic evaluation*" OR "costs and cost analys?s" OR "cost analys?s" OR "return on investment" OR "return to investment" OR "cost effectiveness analys?s" OR "cost-effectiveness analy?s" OR "cost utility analys?s" OR "cost effectiveness evaluation*" OR "cost-effectiveness evaluation*" OR "cost-utility analys?s" OR RCT)
8. **(#1 OR #2) AND (#3 OR #4) AND (#5 OR #6 OR #7)**

6. *List any limits that may apply to your search:*

Gender: Any
Age: 18 years+
Year(s) of publication: 2010 to present
Language(s): English only
Subjects: Humans only

7. *List the databases you will search:*

Academic databases:

i. PsychINFO via Ovid
ii. MEDLINE via Ovid
iii. EMBASE via Ovid
iv. Scopus via Ovid
v. Cochrane Library

Grey literature databases:

i. OpenGrey
ii. WHOLIS (World Health Organisation library database)
iii. APO (Analysis & Policy Observatory)
